# Knockdown of Carboxypeptidase A6 in Zebrafish Larvae Reduces Response to Seizure-Inducing Drugs and Causes Changes in the Level of mRNAs Encoding Signaling Molecules

**DOI:** 10.1371/journal.pone.0152905

**Published:** 2016-04-06

**Authors:** Mark William Lopes, Matthew R. Sapio, Rodrigo B. Leal, Lloyd D. Fricker

**Affiliations:** 1 Programa de Pós-graduação em Bioquímica, Departamento de Bioquímica, Centro de Ciências Biológicas, Universidade Federal de Santa Catarina, Florianópolis, SC, Brazil; 2 Department of Neuroscience, Albert Einstein College of Medicine, Bronx, New York, United States of America; 3 Department of Molecular Pharmacology, Albert Einstein College of Medicine, Bronx, New York, United States of America; University of Modena and Reggio Emilia, ITALY

## Abstract

Carboxypeptidase A6 (CPA6) is an extracellular matrix metallocarboxypeptidase that modulates peptide and protein function by removal of hydrophobic C-terminal amino acids. Mutations in the human *CPA6* gene that reduce enzymatic activity in the extracellular matrix are associated with febrile seizures, temporal lobe epilepsy, and juvenile myoclonic epilepsy. The characterization of these human mutations suggests a dominant mode of inheritance by haploinsufficiency through loss of function mutations, however the total number of humans with pathologic mutations in *CPA6* identified to date remains small. To better understand the relationship between CPA6 and seizures we investigated the effects of morpholino knockdown of *cpa6* mRNA in zebrafish (*Danio rerio*) larvae. Knockdown of *cpa6* mRNA resulted in resistance to the effect of seizure-inducing drugs pentylenetetrazole and pilocarpine on swimming behaviors. Knockdown of *cpa6* mRNA also reduced the levels of mRNAs encoding neuropeptide precursors (*bdnf*, *npy*, *chga*, *pcsk1nl*, *tac1*, *nts*, *edn1*), a neuropeptide processing enzyme (*cpe*), transcription factor (*c-fos*), and molecules implicated in glutamatergic signaling (*grin1a* and *slc1a2b*). Treatment of zebrafish embryos with 60 mM pilocarpine for 1 hour led to reductions in levels of many of the same mRNAs when measured 1 day after pilocarpine exposure, except for *c-fos* which was elevated 1 day after pilocarpine treatment. Pilocarpine treatment, like *cpa6* knockdown, led to a reduced sensitivity to pentylenetetrazole when tested 1 day after pilocarpine treatment. Taken together, these results add to mounting evidence that peptidergic systems participate in the biological effects of seizure-inducing drugs, and are the first *in vivo* demonstration of the molecular and behavioral consequences of *cpa6* insufficiency.

## Introduction

Epilepsy and other seizure disorders are devastating diseases whose onset and genesis remain poorly understood. Many genes have been found to be involved in seizure disorders in humans [1]. The majority of these genes are ion channels that directly contribute to neuronal excitability [1]. A variety of other genes involved in brain development and intercellular signaling are also known to be associated with seizure disorders [1–3] including a recently discovered mutation in the neuropeptide precursor gene *GAL* [4]. However, the mechanism by which mutations in these genes cause a seizure-provoking insult can be complex or indirect. Therefore, research into mechanisms of epileptogenesis is crucial to better understand the cause and genesis of seizures, and ultimately to develop novel diagnostics, predictive models for patient care, and treatment.

Carboxypeptidase A6 (CPA6) is an enzyme in the M14 metallocarboxypeptidase family [5]. It is translated as proCPA6, containing a prodomain which is removed by a furin-like cleavage before secretion [6]. The mature enzyme is secreted from cells and binds to the extracellular matrix (ECM) where it acts as an exopeptidase removing hydrophobic C-terminal amino acids from peptides and proteins [6, 7]. Studies from mice have shown that during development, *Cpa6* mRNA is present in several parts of the brain [8]. In the adult mouse it is most strongly expressed in the olfactory bulb, where it presumably processes neuropeptides secreted from mitral cells, the principal projection neurons [8]. *CPA6* mRNA has also been detected in the hippocampus of adult humans [9]. CPA6 is able to cleave C-terminal hydrophobic residues from a number of brain peptides [7], including some known to regulate neuronal excitability [10, 11]. In some cases, removal of the C-terminal hydrophobic residues produces a form of the neuropeptide that is known to be inactive, while in other cases the CPA6-mediated cleavage converts an inactive peptide into the active form [7].

Two mutations in the *CPA6* gene were originally identified in patients with seizure disorders [9]. Subsequently, additional mutations in *CPA6* have been reported in patients with temporal lobe epilepsy [12] as well as juvenile myoclonic epilepsy [13]. Further, a mother with unexplained epilepsy and a child with epileptic encephelopathy have recently been identified with deleterious *CPA6* mutations [14]. In total, 6 different rare mutations in *CPA6* have been identified in patients with seizure disorders. All of these mutations reduce the levels of active enzyme found in the ECM. It was hypothesized that reduced CPA6 activity in the ECM of patients with *CPA6* mutations led to altered levels of the active forms of neuropeptides, increasing levels of excitatory peptides and/or decreasing levels of inhibitory peptides [15]. Alternatively, it is possible that reduced CPA6 activity in the ECM leads to compensatory changes in expression of proteins involved in neuronal excitability.

To investigate the function of CPA6 in an animal model, we used embryonic zebrafish. Previous work has shown that embryonic zebrafish are a useful model organism to study the behavioral effects of seizure-inducing drugs [16–19]. In the presence of pentylenetetrazole (PTZ), embryonic zebrafish exhibit convulsive behavior and rapid stereotyped motions resembling seizures [16, 17]. When exposed to low concentrations of pilocarpine, embryonic zebrafish increase their total locomotor behavior in a manner similar to mammals [20]. Both PTZ and pilocarpine produce widespread and aberrant electrical discharges in field recordings [17, 21]. These characteristics can be combined with powerful genetic tools such as early embryonic knockdown or overexpression, allowing for the rapid generation of animal models. Zebrafish have a clear ortholog of proCPA6 with high homology to the enzyme found in humans [22]. Like the human ortholog, zebrafish proCPA6 is processed into CPA6 which binds to the ECM and catalyzes the cleavage of C-terminal hydrophobic residues [22]. During larval development, CPA6 is expressed at approximately equal levels from days 1 through 5. This gene is expressed in newly formed somites in the developing larvae, as well as in ectodermal cells of the tail and in a region posterior to the eye [22].

In the present study, morpholino oligonucleotides were used to prevent splicing of *cpa6* transcripts, leading to nonsense mediated decay (i.e. knockdown of *cpa6* mRNA). Zebrafish embryos were studied during the larval stage, after the onset of swimming behavior, and were tested for sensitivity to pentylenetetrazole (PTZ) and pilocarpine. Knockdown of *cpa6* in zebrafish larvae reduced the response to PTZ and pilocarpine without affecting normal motility. To investigate potential mechanisms, the levels of mRNAs encoding signaling molecules were analyzed after *cpa6* knockdown, and after treatment with PTZ or pilocarpine [16, 17, 20, 23]. Several mRNAs were found to be altered by *cpa6* knockdown and by exposure to PTZ and pilocarpine. Taken together, these results suggest a complex interaction of CPA6-mediated extracellular neuropeptide processing and electrical excitability.

## Material and Methods

### 2.1. Zebrafish maintenance and injection of embryos

Zebrafish were maintained under standard conditions at 28°C in the core facility at Albert Einstein College of Medicine as described previously [24, 25]. Embryos were collected from mated pairs and morpholino oligonucleotides and/or mRNAs were injected before the 4 cell stage. Following injection, embryos were maintained at 28°C in egg water [26]. Morpholino oligonucleotides were obtained from Genetools, LLC, and dissolved in distilled water according to manufacturer’s instructions. Sequences of morpholino oligonucleotides are as follows: Ctrl-MO (Genetools standard control): CCTCTTACCTCAGTTACAATTTATA; CPA6-MO2: AGAAAATAAGGGACTCACTGTCAAG; CPA6-MO4: CTTGTCAGCCATGTGCTCACCTCTT. *CPA6* mRNA was generated from a zebrafish CPA6 plasmid using the mMESSAGE mMACHINE kit (Ambion) according to manufacturer’s instructions. In experiments where mRNA and morpholino oligonucleotide were co-injected into embryos, reagents were mixed beforehand and injected as a single bolus. This study was carried out in strict accordance with the recommendations in the Guide for the Care and Use of Laboratory Animals of the National Institutes of Health. The protocol was approved by the Institute for Animal Care and Use Committee of the Albert Einstein College of Medicine (Protocol Number: 20140102).

### 2.2. Quantitation of mRNA levels in zebrafish embryos

Zebrafish embryos were euthanized by addition of sodium hypochlorite (≤7 dpf) or tricaine methane sulfonate (>7 dpf) and stored in RNAlater (Qiagen). Biological replicates of mRNA samples represent pools of mRNA from 6 fish each. Nucleic acids were purified using an RNeasy mini kit (Qiagen), and DNA was digested on the column, prior to elution using an RNAse-Free DNAse Set (Qiagen). Subsequently cDNAs were synthesized from purified RNA products using SuperScript III First Strand Synthesis System (Life Tech) using random hexamers. Purified cDNAs were amplified by quantitative real time PCR using Power SYBR Green with an AB7900 Real Time PCR Instrument (Applied Biosystems). The primers used to amplify PCR products are described in S1 Table.

### 2.3. Assessment of convulsive swimming behavior in zebrafish embryos exposed to pentylenetetrazole (PTZ)

Embryonic zebrafish 3 or 7 days post-fertilization (dpf) were placed in 12-well cluster plates and behavioral experiments were carried out at 30–32°C. Temperatures were maintained using an incubator and temperatures were checked regularly. Behaviors were recorded using a JVC camcorder (model GZ-MS230AU). Ten minutes of baseline activity were collected and analyzed with embryo medium alone. An equal volume of embryo medium containing 5 or 30 mM of PTZ was added to each well to reach a final concentration of 2.5 or 15 mM of PTZ, respectively. Subsequently, behaviors after the addition of drug were recorded for 18 minutes. Videos were scored to quantify abnormal convulsive swimming behaviors in zebrafish embryos according to established criteria [16, 17, 27]. Convulsive behaviors were recorded for each minute interval and occurred with more frequency with 15 mM PTZ than with 2.5 mM PTZ. Behavioral scoring of videos was done by observers blinded with respect to experimental treatment of the animals.

### 2.4. Assessment of locomotion in zebrafish embryos exposed to pilocarpine

Zebrafish were placed in 12-well cluster plates, as described above for the studies on PTZ. Ten minutes of baseline activity were collected with embryo medium alone. One and one half minutes were analyzed (8.5–10.0 min) during the baseline. An equal volume of embryo medium containing 30, 60 or 120 mM of pilocarpine was added to each well to reach a final concentration of 15, 30 or 60 mM of pilocarpine, respectively. Subsequently, behavior was recorded for 18 minutes. One and one half minutes were analyzed (5.0–6.5 min) after pilocarpine exposure. Videos were scored to quantify number of quadrant transitions (crossings) similar to previously validated work [20]. Behavioral scoring of videos was done by observers blinded with respect to experimental treatment of the animals.

### 2.5. Assays for motility and responsiveness to stimuli

Three dpf zebrafish embryos were placed into individual wells of a 12-well cluster plate in 1x embryo medium. A stimulus was delivered by pipetting 200 μl of embryo medium towards them at mild force. The stimulus was calibrated so that it would evoke a response ~40% of the time under basal conditions. Subsequently 1 volume of embryo medium containing 5 mM PTZ was added for a final concentration of 2.5 mM PTZ, and the assay was repeated at 5 minute intervals. Ten stimuli were delivered to a fish at each time point. A similar assay was performed with a tactile stimulus produced by touching zebrafish embryos lightly with a hair. The stimulus was calibrated so that it would evoke a response ~80% of the time under basal conditions. 3 stimuli were delivered to a fish, and responses were averaged.

### 2.6. Statistical analysis

The data were checked for normality of frequency distribution with the Kolmogorov-Smirnov test and expressed as mean ± S.E.M. Statistical analyses were carried using Student’s *t* test for independent samples, one-way or two-way ANOVA, and repeated measures ANOVA followed by Tukey HSD multiple range tests when appropriate. The accepted level of significance for the tests was *p* ≤ 0.05. Parametric tests were performed using the Statistica software package (StatSoft Inc., Tulsa, OK, USA).

## Results

### 3.1. Morpholino-mediated knockdown of *cpa6* reduces the behavioral response to PTZ or pilocarpine in 3dpf zebrafish embryos

**Fig 1:**
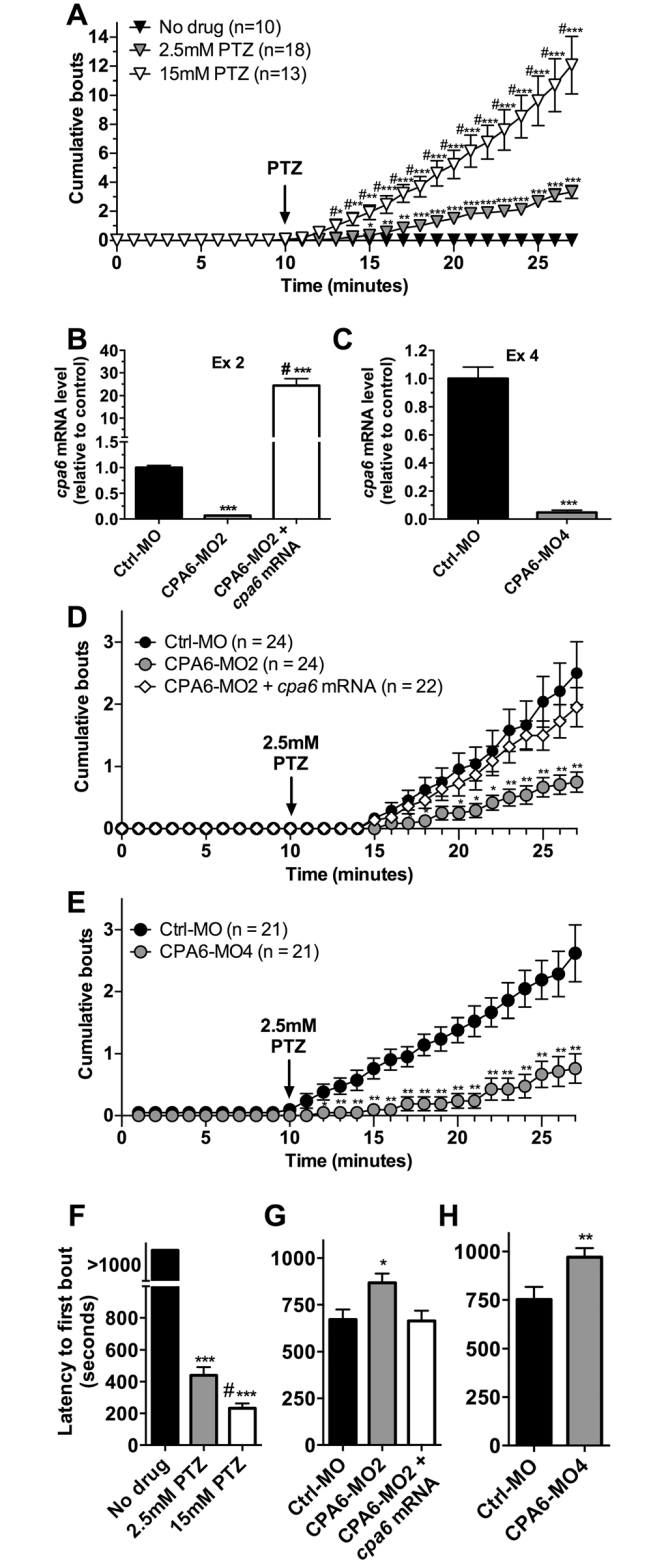
Knockdown of *cpa6* reduces PTZ-evoked convulsive swimming behavior in 3 dpf zebrafish embryos. (A) Zebrafish were tested for convulsive swimming behavior in the presence of 2.5 or 15 mM PTZ. After a 10 minute baseline period without drug, 2.5 or 15 mM PTZ were added to the bath and convulsive swimming behavior was recorded by an observer blinded to the treatment groups, using previously established criteria [16, 17]. Statistical analysis was performed by repeated measures ANOVA, followed by Tukey HSD: *, *p* < 0.05; **, *p* < 0.01; ***, *p* < 0.001 compared with No drug group in respective time points; #, *p* < 0.05 compared with 2.5 mM PTZ group in respective time points. (B) Quantitative PCR revealed near total knockdown in the targeted exon for CPA6-MO2, while co-injection of CPA6-MO2 and 500 pg mRNA increased transcript levels of *cpa6* by ~25 fold (n = 4). One-way ANOVA, followed by Tukey HSD: ***, *p* < 0.001 compared with Ctrl-MO group; #, *p* < 0.001 compared with CPA6-MO2 group. (C) Quantitative PCR revealed near-total knockdown of the targeted exon for CPA6-MO4 (n = 4). Student’s *t* test: ***, *p* < 0.001 compared with Ctrl-MO group. (D) CPA6-MO2 injected animals exposed to 2.5 mM PTZ showed fewer bouts of convulsive swimming behavior relative to Ctrl-MO and this behavior was rescued by co-injection of CPA6-MO2 and *cpa6* mRNA. Repeated measures ANOVA, followed by Tukey HSD: *, *p* < 0.05; **, *p* < 0.01 compared with Ctrl-MO group in respective time points. (E) CPA6-MO4 injected animals showed fewer bouts of convulsive swimming behavior relative to Ctrl-MO. Repeated measures ANOVA, followed by Tukey HSD: *, *p* < 0.05; **, *p* < 0.01 compared with Ctrl-MO group in respective time points. (F) The “no drug” control group of zebrafish embryos displayed no bouts of convulsive swimming behavior, resulting in a latency greater than the testing period (1080 seconds). Latency to the first bout was decreased in zebrafish embryos exposed to 2.5 and 15 mM PTZ. One-way ANOVA, followed by Tukey HSD: ***, *p* < 0.001 compared with No drug group; #, *p* < 0.05 compared with 2.5 mM PTZ group. (G, H) CPA6-MO2 and -MO4 injected embryos exposed to 2.5 mM PTZ had a higher latency to the first bout of convulsive swimming behavior when compared to fish injected with control morpholino. In panels G and H, the y-axis is similar to that of panel F. Co-injection of *cpa6* mRNA along with CPA6-MO2 blocked the effect of the morpholino oligonucleotide. One-way ANOVA, followed by Tukey HSD used in panel G: *, *p* < 0.05 compared with Ctrl-MO and CPA6-MO2 + *cpa6* mRNA groups. Student’s t test used in panel H: **, *p* < 0.01 compared with Ctrl-MO group. Error bars show SEM.

Zebrafish embryos have previously been established as a model system for examining behavior in response to seizure-inducing drugs [16, 17]. In response to addition of PTZ into the bath water, the 3 dpf fish respond with increased swimming behavior in a dose and time-dependent fashion (Fig 1). In the first minutes after addition of low doses of PTZ, locomotion is elevated [17]. At higher doses and/or longer exposures, convulsive behaviors have been described in which the fish show rapid circular stereotyped swimming behaviors or uncoordinated movements [17]. Wild type 3 dpf zebrafish embryos were treated with PTZ for 18 minutes and convulsive swimming behavior was recorded for each minute interval, using criteria described previously [18]. Repeated measures ANOVA revealed a significant effect due to PTZ exposure [*F*(2,34) = 22.63, *p* < 0.001]. Wild type animals exposed to concentrations of 2.5 or 15 mM PTZ showed significantly more bouts of convulsive swimming behavior, with 2.5 mM producing approximately 3 bouts and 15 mM PTZ producing 12 bouts over the 17 minute observation period (Fig 1A).

CPA6-MO2 targets the exclusion of exon 2 of the *cpa6* mRNA transcript, while CPA6-MO4 targets exon 4 (S1 Fig). Action of either morpholino is predicted to cause a frameshift in the transcript. Primer sets were designed to amplify exons 2 and 4 of the *cpa6* transcript in zebrafish embryos. For both CPA6-MO2 and CPA6-MO4, one-way ANOVA [*F*(2,9) = 63.20, *p* < 0.001] and Student’s *t* test (*p* < 0.001), respectively, revealed a significant effect; the transcript levels were reduced ~90% for the targeted exon (Fig 1B and 1C). In order to overexpress CPA6 in zebrafish, 500 or 1000 pg of *cpa6* mRNA were injected into 1–4 cell stage embryos. One-way ANOVA revealed a significant effect [*F*(2,14) = 27.30, *p* < 0.001]; the *cpa6* mRNA injection caused a ~20 and ~40-fold overexpression of *cpa6* transcripts, respectively at 3 dpf (S2C Fig). In zebrafish embryos co-injected with CPA6-MO2 and *cpa6* mRNA, one-way ANOVA also revealed a significant effect [*F*(2,9) = 63.20, *p* < 0.001]); the transcript levels of *cpa6* were increased ~25 times (Fig 1B).

Repeated measures ANOVA revealed a significant effect in injected embryos exposed to 2.5 mM PTZ [*F*(2,24) = 4.26, *p* < 0.001]; CPA6-MO2 had markedly fewer bouts of convulsive swimming behavior than Ctrl-MO injected embryos exposed to the same concentration of PTZ (Fig 1D). The response to PTZ was rescued by co-injection of *cpa6* mRNA with CPA6-MO2 (Fig 1D). The overexpression of *cpa6* mRNA levels alone (i.e. in embryos not treated with the CPA6 morpholino) did not affect the number of bouts of convulsive swimming behavior induced by PTZ (repeated measures ANOVA, [*F*(2,34) = 1.32, *p* = 0.10]), (S2A Fig). Similarly, repeated measures ANOVA revealed a significant effect [*F*(1,26) = 12.25, *p* < 0.001]; CPA6-MO4 injected embryos exposed to 2.5 mM PTZ showed a decrease in cumulative bouts of convulsive swimming behavior, compared to the Ctrl-MO (Fig 1E). The significant difference in PTZ-induced behavior between the CPA6-MO4 and Ctrl-MO-injected embryos was not observed when 15 mM PTZ was used, suggesting saturation of the effect at the higher dose of PTZ (repeated measures ANOVA, [*F*(2,34) = 0.84, *p* = 0.72]) (S3 Fig).

Latency to first bout of convulsive swimming behavior was examined as described previously [18]. The untreated control group showed no bouts of convulsive swimming behavior (Fig 1F) over the duration of the testing period (1080 seconds). One-way ANOVA revealed a significant effect [*F*(2,38) = 87.99, *p* < 0.001]; the latency to first bout was decreased in zebrafish embryos exposed to 2.5 and 15 mM PTZ (Fig 1F). In parallel with the results shown in Fig 1D and 1E, one-way ANOVA [*F*(2,67) = 4.82, *p* < 0.05] and Student’s t test (*p* < 0.01) revealed a significant effect; CPA6-MO2 and CPA6-MO4 injected animals exposed to 2.5 mM PTZ had a higher latency to first bout compared to Ctrl-MO injected animals (Fig 1G and 1H). Co-injection of *Cpa6* mRNA with CPA6-MO2 restored the response to that of Ctrl-MO injected embryos (Fig 1G).

**Fig 2:**
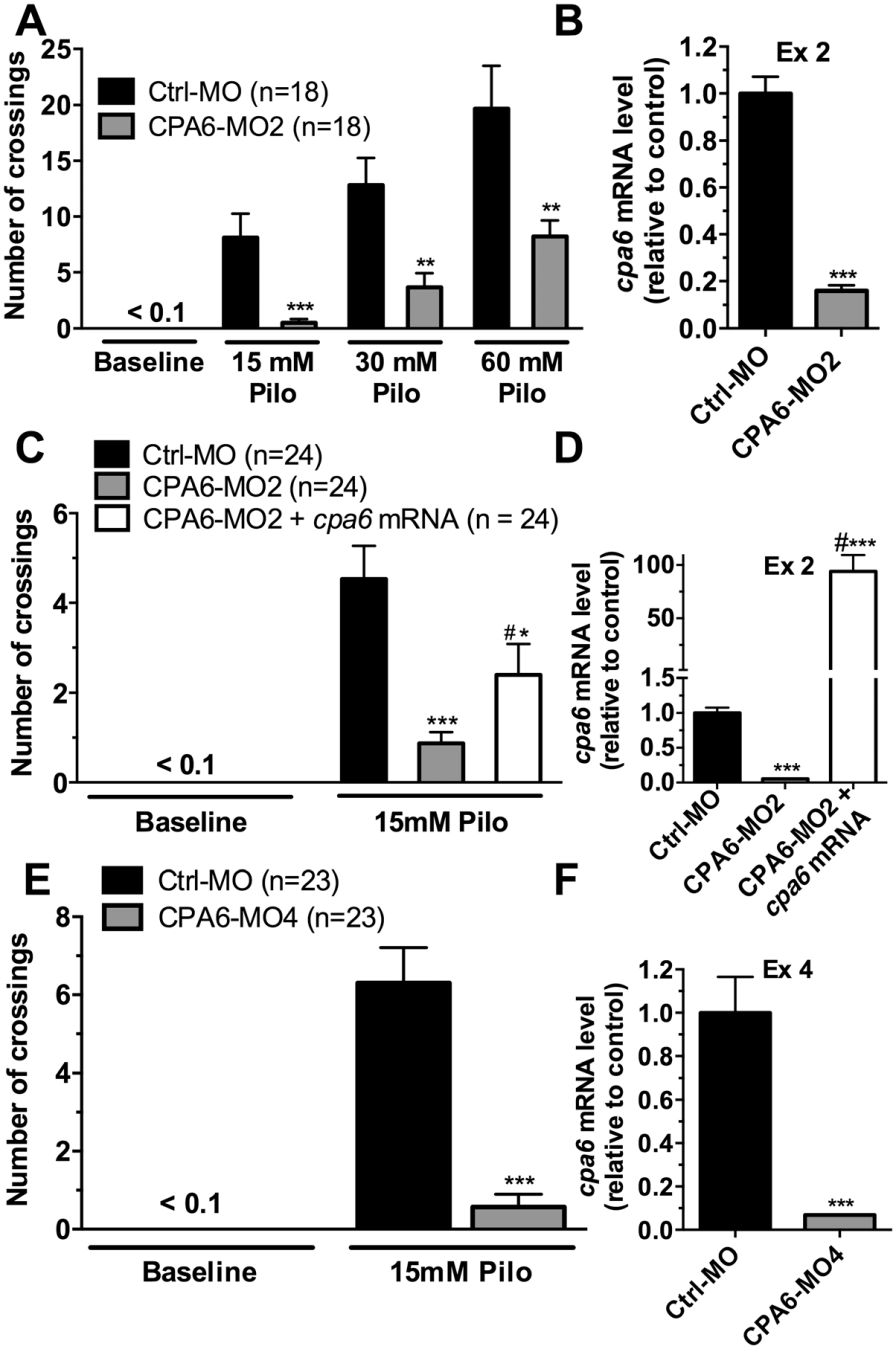
Knockdown of *cpa6* reduces pilocarpine-induced increase in locomotion in 3 dpf zebrafish embryos. (A) Animals were placed in a 12-well cluster plate and tested for number of crossings between quadrants (locomotor activity) in the presence of 15, 30 or 60 mM pilocarpine. After a 10 minute baseline period, pilocarpine was added to the bath to produce a final concentration of 15, 30 or 60 mM. The number of crossings was recorded over a 1.5 minute period by an observer blinded to the treatment group; for baseline the period from 8.5–10.0 min was analyzed, and for pilocarpine the period from 5.0–6.5 min after drug treatment was analyzed. The number of crossings was increased by pilocarpine, with a greater increase in zebrafish embryos injected with Ctrl-MO compared to CPA6-MO2. Statistical analysis was performed by two-way ANOVA, followed by Tukey HSD: **, *p* < 0.01; ***, *p* < 0.001 compared with respective Ctrl-MO group. (B) Quantitative PCR revealed near total knockdown in the targeted exon for CPA6-MO2 (n = 4). Student’s *t* test: ***, *p* < 0.001 compared with Ctrl-MO group. (C) CPA6-MO2 injected animals exposed to 15 mM pilocarpine showed decreased number of crossings relative to Ctrl-MO and this effect was partially rescued by co-injection of 500 pg *cpa6* mRNA along with the CPA6-MO2. One-way ANOVA, followed by Tukey HSD: *, *p* < 0.05; ***, *p* < 0.001 compared with Ctrl-MO group; #, *p* < 0.05 compared with CPA6-MO2 group. (D) Quantitative PCR revealed near total knockdown of the targeted exon for CPA6-MO2, while co-injection of *cpa6* mRNA along with CPA6-MO2 increased the transcript levels of *cpa6* (n = 4). One-way ANOVA followed by Tukey HSD: ***, *p* < 0.001 compared with Ctrl-MO group; #, *p* < 0.001 compared with CPA6-MO2 group. (E) CPA6-MO4 injected animals exposed to 15 mM pilocarpine showed decreased number of crossings relative to Ctrl-MO (n = 4). Student’s *t* test: ***, *p* < 0.001 compared with Ctrl-MO group. (F) Quantitative PCR revealed near total knockdown in the targeted exon for CPA6-MO4. Student’s *t* test: ***, *p* < 0.001 compared with Ctrl-MO group. Error bars show SEM.

Pilocarpine induces seizures in vertebrates including zebrafish [20, 23, 28–30]. At low doses, pilocarpine increases the locomotor activity of zebrafish embryos, which can be used as an index of stimulation of these animals. Locomotor activity was measured by counting quadrant transitions (number of crossings). Track-length was also examined, but was less reliable as a measure of activity (S4 Fig). Zebrafish embryos were analyzed for baseline behavior and after pilocarpine. Two-way ANOVA revealed a significant effect [*F*(2,100) = 9.70, *p* < 0.001]; pilocarpine increased locomotor behavior in a dose-dependent manner (Fig 2A). Embryos injected with CPA6-MO2 showed a reduced response to all three doses of pilocarpine, relative to control morpholino-injected embryos (Fig 2A). The effect caused by CPA6-MO2 injection (one-way ANOVA [*F*(2,69) = 8.72, *p* < 0.001]) was partially rescued by co-injection with *cpa6* mRNA, as indicated by post hoc test (Fig 2C). In the absence of CPA6 morpholino, overexpression of *cpa6* mRNA levels did not affect the increase in locomotion induced by pilocarpine (S2B Fig). Embryos injected with CPA6-MO4 showed a reduced response to pilocarpine exposure (Student’s *t* test, *p* < 0.001) (Fig 2E). For both CPA6-MO2 and CPA6-MO4, one-way ANOVA [*F*(2,9) = 39.37, *p* < 0.001] and Student’s *t* test (*p* < 0.001), respectively, revealed a significant effect; the transcript levels were reduced ~90% for the targeted exon (Fig 2D and 2F). In zebrafish embryos co-injected with CPA6-MO2 and *cpa6* mRNA, one-way ANOVA also revealed a significant effect [*F*(2,9) = 39.37, *p* < 0.001]); the transcript levels were increased, as indicated by post hoc test (Fig 2D).

Zebrafish embryos injected with Ctrl-MO showed ~4-5-fold more bouts of convulsive swimming behavior in response to 2.5 mM PTZ when tested at 7 dpf compared to 3 dpf (S5 Fig). Even though the baseline of convulsive swimming behavior in response to 2.5 mM PTZ was higher at 7 dpf, repeated measures ANOVA revealed a significant effect [*F*(2,34) = 16.01, *p* < 0.001]; the zebrafish embryos injected with CPA6-MO2 and MO4 had markedly fewer bouts of convulsive swimming behavior compared with Ctrl-MO injected animals (S5 Fig). Zebrafish embryos injected with CPA6-MO2 or CPA6-MO4 showed a significant reduction in *cpa6* levels at 7 dpf (Student’s *t* test, *p* < 0.01), (S5 Fig).

### 3.2. Knockdown of *cpa6* mRNA does not affect motility and responsiveness to stimuli of zebrafish embryos

**Fig 3:**
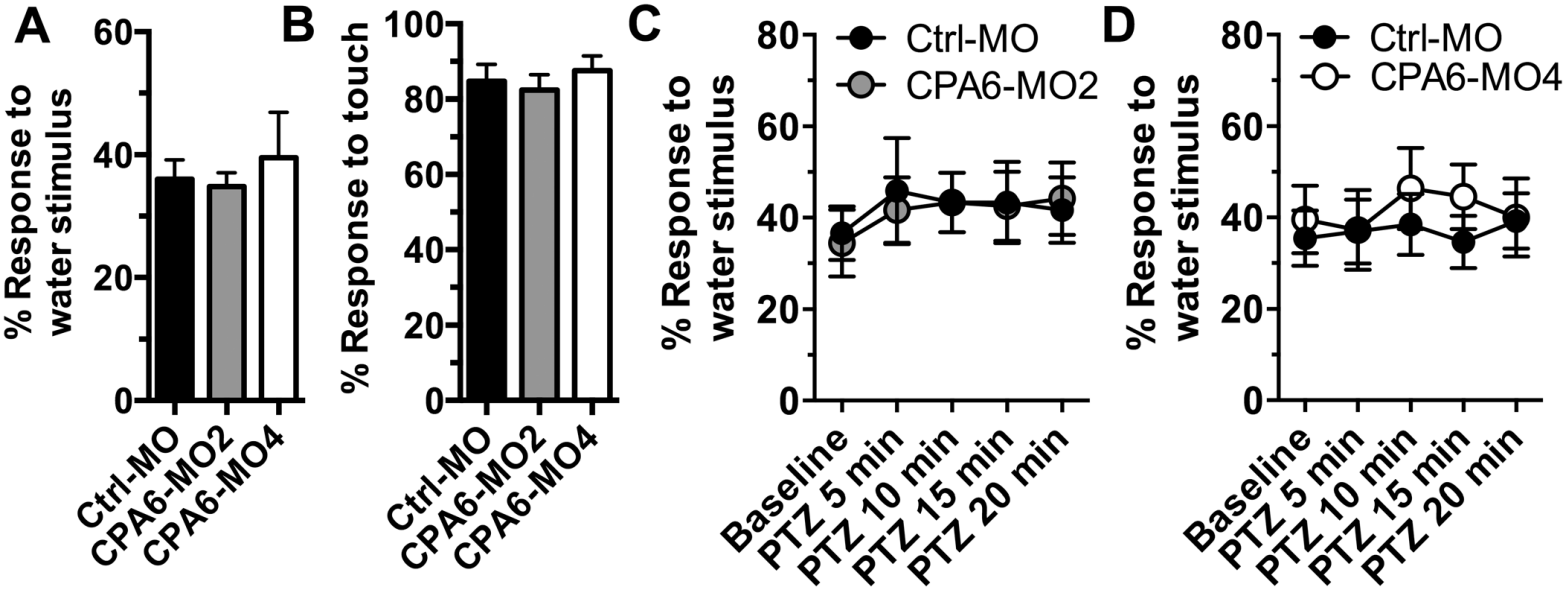
Zebrafish embryos injected with CPA6-MO2 and -MO4 are responsive to stimuli. Zebrafish embryos 3 dpf were stimulated by pushing 200 μl of the embryo media in which they were suspended towards them using a P-200 pipette at moderate pressure (A) or by touching with a hair (B). Water stimuli were calibrated to evoke a response only ~40% of the time under basal conditions while touch stimuli evoked a response 80% of the time. Statistical analysis was performed by one-way ANOVA. (C,D) PTZ (2.5 mM) was added to the embryo media and fish were tested at 5 minute intervals for responsiveness to the water stimulus. There were no statistically significant differences between embryos injected with Ctrl-MO, CPA6-MO2 or CPA6-MO4 either in basal conditions or in the presence of PTZ during the time periods examined for either stimulation paradigm. Statistical analysis was performed by repeated measures ANOVA. Error bars show SEM.

Convulsive swimming behavior was defined according to previous standards described in fish exposed to seizure-inducing drugs [16, 17]. However, fish that have tail defects or are otherwise unable to behave normally may be able to move, or may move in different patterns. Although CPA6-MO2 and CPA6-MO4 injected zebrafish had no obvious physical abnormality, we tested whether they could respond to stimuli under basal conditions and in the presence of PTZ. Knockdown of *cpa6* had no effect on response to stimulation with embryo medium under basal conditions (one-way ANOVA [*F*(2,44) = 0.24, *p* = 0.79]) (Fig 3A). In the presence of PTZ, repeated measures ANOVA also revealed no significant change on response to stimulation (CPA6-MO2, [*F*(1,4) = 0.91, *p* = 0.46] and CPA6-MO4, [*F*(1,4) = 0.43, *p* = 0.78]) (Fig 3C and 3D, respectively). Tactile responsiveness was tested by touching the zebrafish embryos with a hair, as described previously [18]. No difference in response to tactile stimulation was observed between control and *cpa6* knockdown animals in basal conditions (one-way ANOVA [*F*(2,81) = 0.37, *p* = 0.69]) (Fig 3B).

### 3.3. Transcriptional effects of *cpa6* knockdown and treatment with seizure-inducing drugs

Because CPA6 is an extracellular peptide-processing enzyme [22], the effect of *cpa6* knockdown on susceptibility to seizure-inducing drugs could be due to altered synaptic levels of neuropeptides. It is also possible that the knockdown of *cpa6* causes secondary changes in mRNA expression, possibly due to altered synaptic peptide levels. Therefore, we investigated the mRNA levels of potential substrates of CPA6 as well as several genes known to be regulated by neuronal activity or contribute to epilepsy in mammals [29, 30].

**Fig 4:**
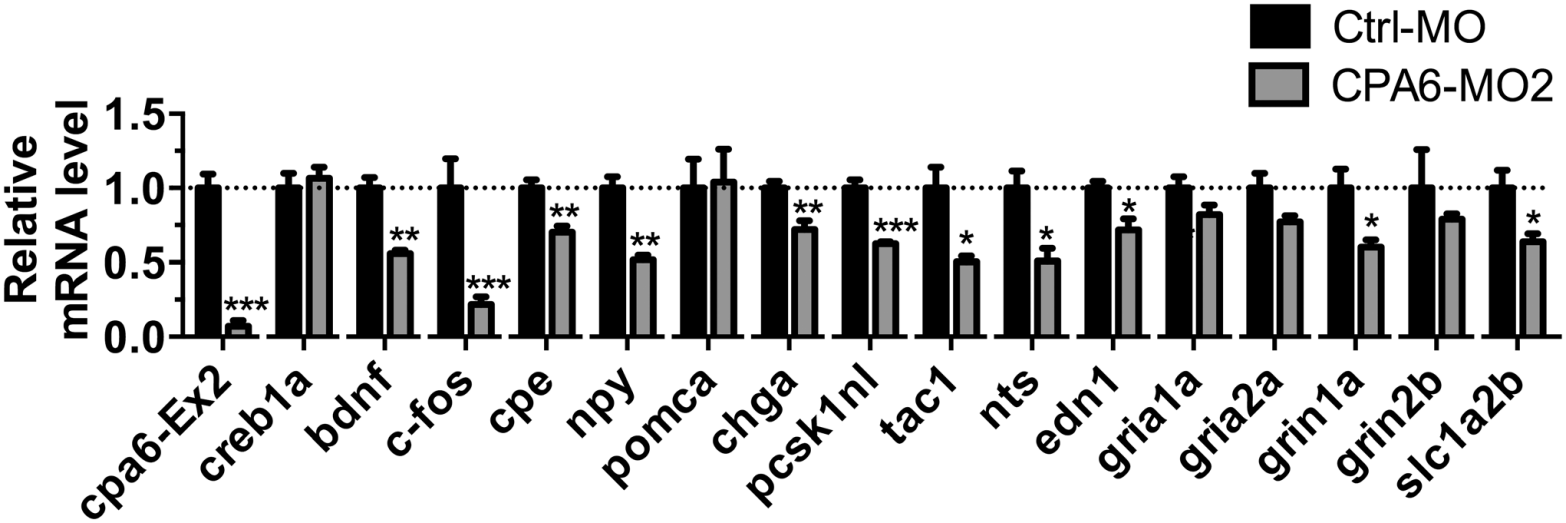
Knockdown of *cpa6* mRNA affects levels of other mRNAs in 3 dpf zebrafish embryos. Ctrl-MO or CPA6-MO2 were injected into embryo yolks before the 4-cell stage. At 3 dpf, zebrafish embryos were collected, mRNA extracted and quantitative PCR performed to measure levels of mRNA encoding neuropeptides, neuropeptide processing enzymes, transcription factors, and proteins involved in glutamate signaling or transport. Error bars show SEM (n = 4 for each group). Statistical analysis was performed by Student’s *t* test: *, *p* < 0.05; **, *p* < 0.01; ***, *p* < 0.001 compared with Ctrl-MO group.

In *cpa6* knockdown animals, Student’s *t* test revealed significant changes in mRNA levels of *cpa6* and the transcription factor *c-fos* (Fig 4). These animals also showed a decrease in mRNA levels for the precursors of several neuropeptides or other secreted peptides (Fig 4) including brain-derived neurotrophic factor (*bdnf*), neuropeptide Y (*npy*), chromogranin A (*chga*), proSAAS (*pcsk1nl*), protachykinin 1 (*tac1*), neurotensin (*nts*), and endothelin 1 (*edn1*). Carboxypeptidase E (*cpe*), a secretory vesicle neuropeptide-processing enzyme that converts neuropeptide-intermediates into the mature form, was also decreased by *cpa6* knockdown. Statistically significant decreases were also observed for mRNA levels of *grin1a* (glutamate receptor ionotropic, N-methyl D-aspartate 1a) and *slc1a2b* (solute carrier family 1, glial high affinity glutamate transporter, member 2b) (Fig 4).

**Fig 5:**
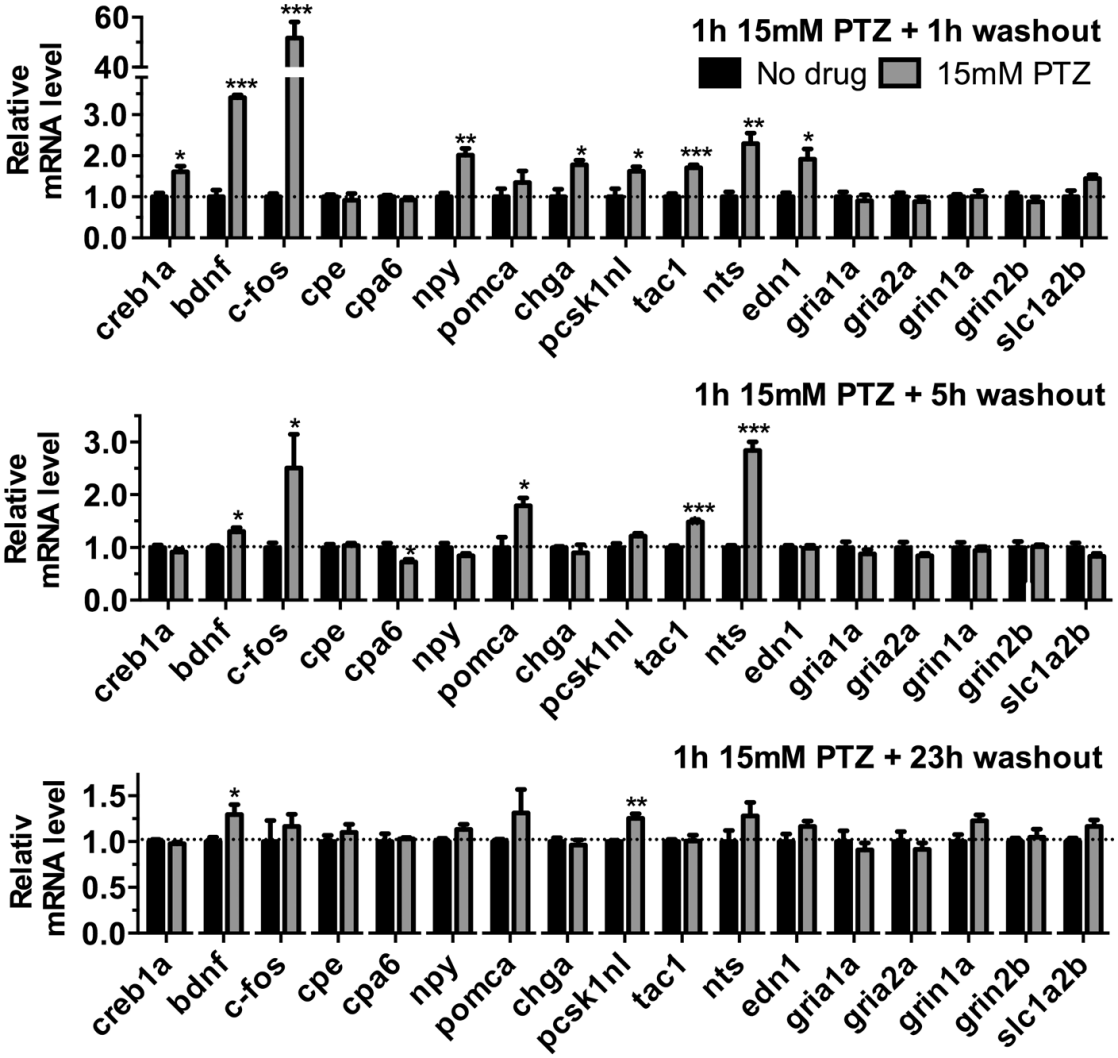
PTZ treatment affects mRNA levels in zebrafish embryos. Zebrafish embryos (wild-type 3 dpf) were exposed to 15 mM PTZ for 1 h and then transferred to drug-free embryo medium for 1, 5, or 23 h after which mRNA was extracted. Quantitative PCR was used to examine levels of various mRNAs. Error bars show SEM (n = 4 for each group). Statistical analysis was performed by Student’s *t* test: *, *p* < 0.05; **, *p* < 0.01; ***, *p* < 0.001 compared with Ctrl-MO group.

To test if the mRNAs examined in Fig 4 were affected by treatment of zebrafish embryos with seizure-inducing drugs, wild type fish were exposed to 15 mM PTZ for 1 h and mRNA levels of targets were analyzed 1, 5 and 23 h after the end of PTZ exposure (i.e. 2, 6, and 24 h after the start of PTZ exposure). At 1 hour after PTZ exposure, Student’s *t* test revealed significant changes in mRNA levels of *creb1a*, *bdnf*, *c-fos*, *npy*, *chga*, *pcsk1nl*, *tac1*, *nts* and *edn1*, which were each up-regulated compared with untreated controls (Fig 5). Five hours after PTZ exposure, the mRNA levels of *bdnf*, *c-fos*, *pomca*, *tac1* and *nts* were increased while the level of *cpa6* mRNA was decreased relative to untreated controls (Fig 5). One day after PTZ exposure, only *bdnf* and *pcsk1nl* mRNA levels remained significantly increased (Fig 5). There were no significant changes in mRNA levels of the glutamate receptors or transporters examined in our study at any time point after PTZ exposure.

**Fig 6:**
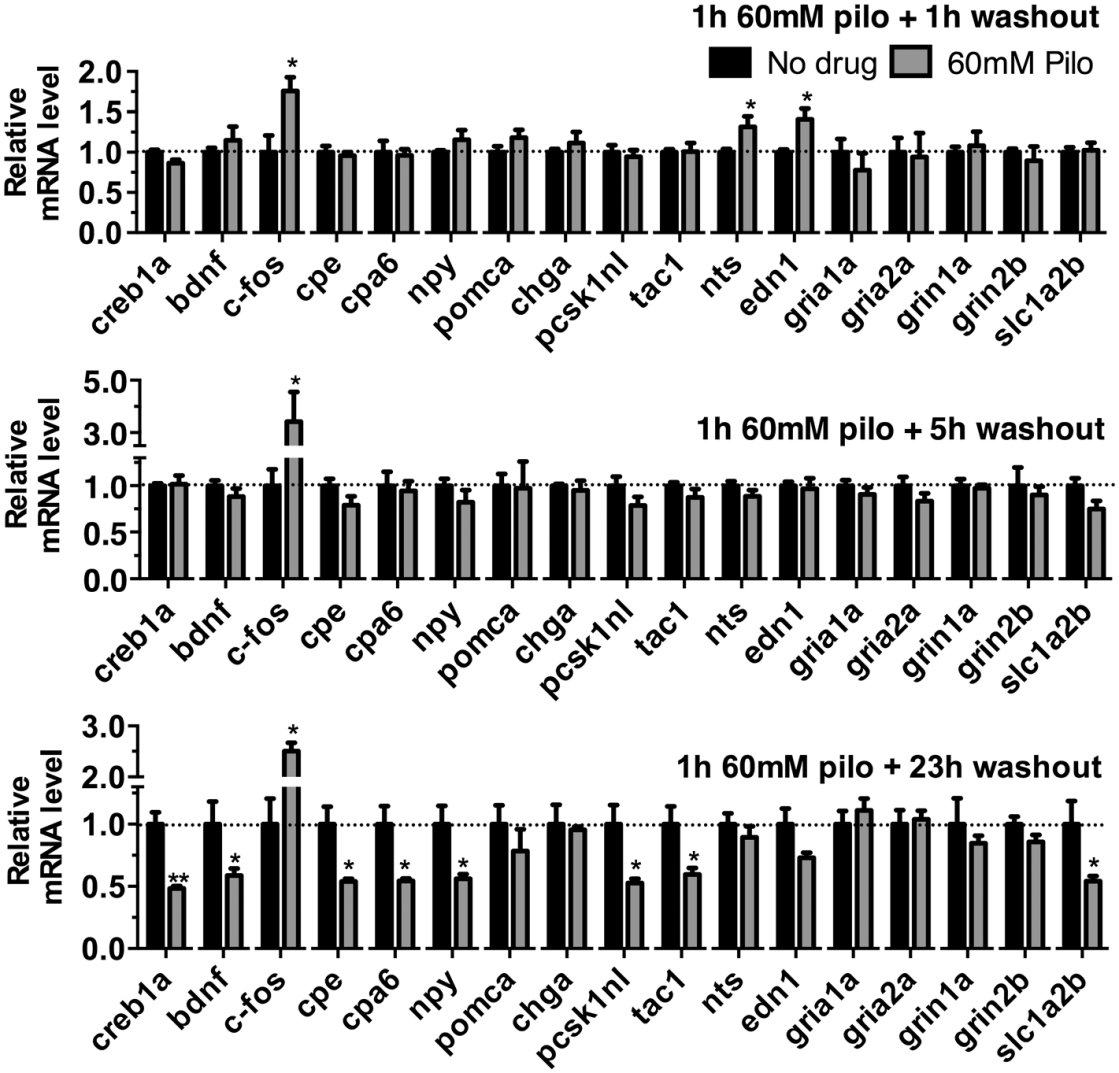
Effects of pilocarpine on mRNA levels of signaling molecules in 3 dpf zebrafish embryos. Wild-type zebrafish embryos were exposed to 60 mM pilocarpine for 1 h, the fish were transferred to drug-free embryo medium, and mRNA was extracted after 1, 5, or 23 h washout without drug. Quantitative PCR was used to measure mRNA levels of targets. Error bars show SEM (n = 4 for each group). Statistical analysis was performed by Student’s *t* test: *, *p* < 0.05; **, *p* < 0.01 compared with respective Ctrl-MO group.

We also tested the effects of pilocarpine in zebrafish embryos. Wild type fish were exposed to 60 mM pilocarpine for 1 h and mRNA levels of targets analyzed 1, 5 and 23 h after pilocarpine exposure. Student’s *t* test revealed significant changes, one hour after pilocarpine exposure, the mRNA levels for *c-fos*, *nts* and *edn1* were up regulated when compared with untreated controls (Fig 6). Five hours after pilocarpine exposure, only *c-fos* mRNA levels were significantly increased (Fig 6). One day after pilocarpine exposure, *c-fos* remained elevated while mRNA levels for *bdnf*, *npy*, *pcsk1nl* and *tac1* were decreased (Fig 6). Levels of *cpe*, *cpa6* and *creb1a* mRNA were also decreased (Fig 6). Levels of mRNAs encoding glutamate receptors were not significantly altered, but the glutamate transporter *slc1a2b* mRNA was significantly decreased (Fig 6).

### 3.4. Pretreatment with 60 mM pilocarpine reduces behavioral response to 2.5 mM PTZ

**Fig 7:**
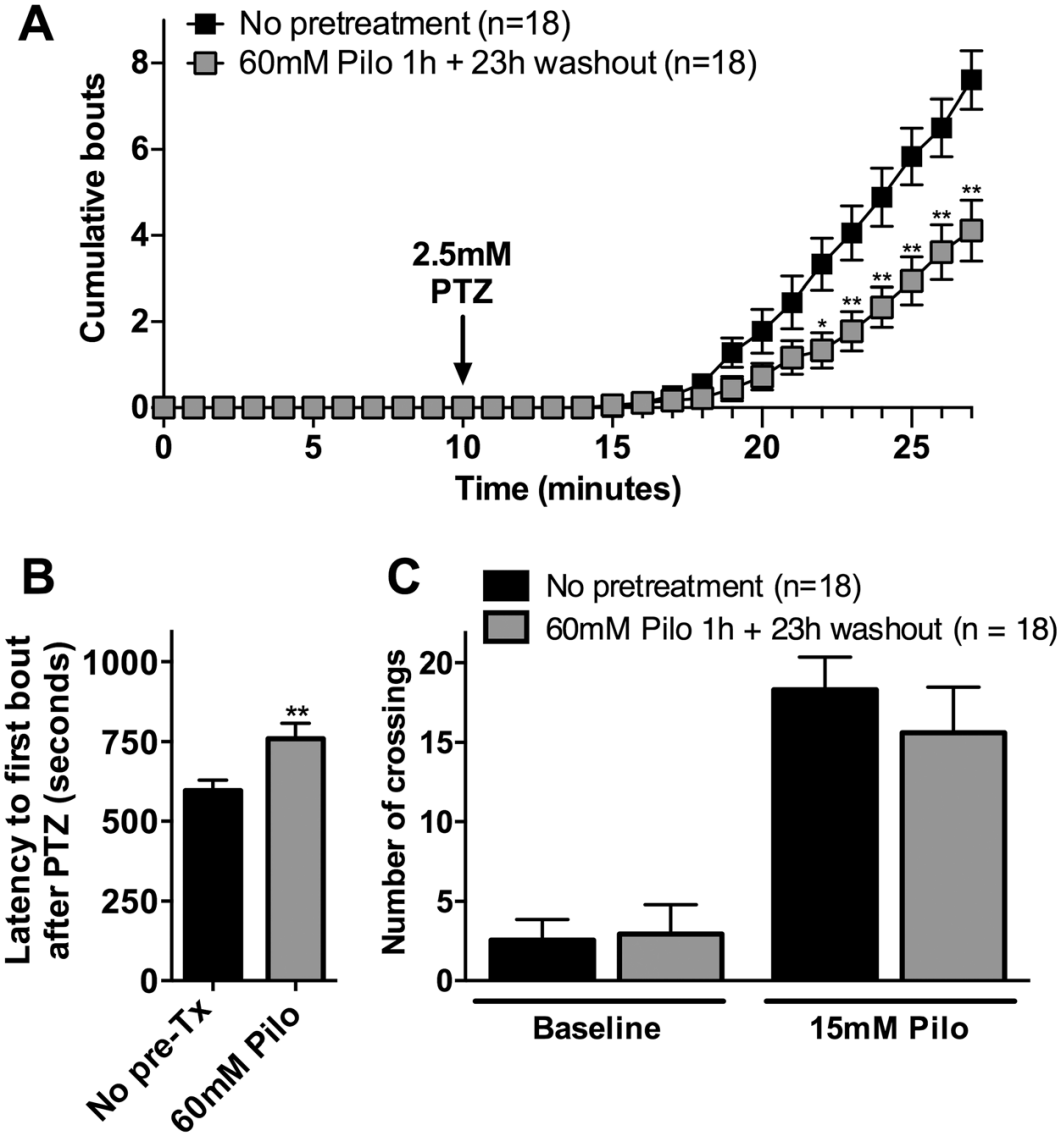
The response of 4 dpf zebrafish embryos to PTZ and pilocarpine is affected by a 1 h exposure to 60 mM pilocarpine at 3 dpf. Wild-type zebrafish embryos were divided into two groups. After 3 dpf, one group was exposed to 60 mM pilocarpine for 1 h while the other group was not treated with drug. Following an additional 23 h period, the 4 dpf zebrafish were examined for convulsive swimming behavior by an investigator blinded to the treatment groups. (A) In the absence of additional drug, no bouts of convulsive swimming behavior were observed over a 10 minute period for either unexposed or pilocarpine-exposed animals. When treated with 2.5 mM PTZ for 17 minutes, convulsive swimming behavior was observed. The group previously exposed to pilocarpine showed fewer bouts than the control group. Statistical analysis was performed by repeated measures ANOVA, followed by Tukey HSD: *, *p* < 0.05; **, *p* < 0.01 compared with No pretreatment group in respective time points. (B) Latency to the first bout was measured after 2.5 mM PTZ exposure. Pre-treatment with 60 mM pilocarpine increased the latency to first bout: Student’s *t* test. **, *p* < 0.01 compared with no pretreatment group. (C) Animals were tested for number of crossings (locomotor activity) under baseline conditions and in response to 15 mM pilocarpine. No significant differences in number of crossings were observed in the group previously exposed to 60 mM pilocarpine, relative to control group; both groups showed a comparable increase in locomotion in response to 15 mM pilocarpine. Statistical analysis was performed by Student’s *t* test. Error bars show SEM.

Based on the observation that many of the mRNAs altered by *cpa6* knockdown were also altered 1 day following 60 mM pilocarpine treatment, we examined whether animals treated with pilocarpine showed reduced sensitivity to PTZ-induced convulsive swimming behavior, as found for the *cpa6* knockdown. The behavioral response of animals to 2.5 mM PTZ was assessed 23 hours after 1 hour treatment with 60 mM pilocarpine. Repeated measures ANOVA revealed a significant effect [*F*(1,12) = 9.13, *p* < 0.001]; zebrafish embryos pre-treated with pilocarpine and then exposed to 2.5 mM PTZ for 18 minutes showed a decrease in the cumulative bouts (Fig 7A) and an increase in latency to first bout compared to untreated controls (Student’s *t* test, *p* < 0.01), (Fig 7B). These behavioral effects were similar to those caused by injection of CPA6-MO2 or CPA6-MO4. Pretreatment with 60 mM of pilocarpine had no effect on convulsive swimming behavior induced by 15 mM pilocarpine (Fig 7C).

## Discussion

Aberrant conductance through ion channels, which exerts direct control over electrical activity, has long been known to underlie many genetic epilepsies. Peptidergic signaling through neuropeptide receptors can alter neuronal excitability by diverse mechanisms, including the modulation of expression and/or conductance of ion channels. A number of peptidergic signaling pathways as well as peptidergic cell populations are altered in epilepsy, and can change the outcome of epilepsy in humans [31–33]. Evidence from several studies suggests that peptides are powerful signaling molecules with regards to regulation of seizures [31, 32, 34, 35].

CPA6 is an extracellular enzyme that can process neuropeptides by removal of one or more amino acids [6]. In the case of enkephalin, CPA6 removes hydrophobic C-terminal residues which would inactivate the peptide by preventing its binding to the opioid receptors. However, in the case of angiotensin I, CPA6 removes hydrophobic residues and converts the peptide into angiotensin II, the form that is active towards the angiotensin receptors. Thus, CPA6 can potentially either inactivate or activate different neuropeptides *in vitro* [6]. Given the relationship between *CPA6* mutations and epilepsy, studies to elucidate the substrates of CPA6 could shed new light on epileptogenesis. *CPA6* mRNA is expressed mainly in the olfactory bulb in the adult mouse and human [8, 36, 37] with a broader pattern of expression throughout development [8]. In adult animals, it is likely that CPA6 processes neuropeptides at the nerve terminals of olfactory bulb peptidergic projection neurons to the piriform cortex or entorhinal cortex, both of which can trigger seizures in the presence of abnormal stimulation [38, 39]. Low but detectable levels of *CPA6* mRNA are present in human hippocampus, nucleus raphe, and cortex [9], and it is possible that CPA6 activity in these regions contributes to electrical excitability.

In order to further understand the role of CPA6 in the regulation of neuronal activity, animal models of CPA6 deficiency are needed. To this end, we generated a *cpa6* knockdown zebrafish model. The present study builds on a growing literature establishing the use of zebrafish to study genes that modulate the response to seizure-inducing drugs [16, 17, 19]. Our finding that knockdown of *cpa6* reduces responsiveness to two different seizure-inducing stimulants (Figs 1 and 2) with no concomitant alteration in motility (Fig 3) demonstrates that the effect of *cpa6* knockdown is selective to the response to the drugs, and not the result of a general change in motility. Additionally, the finding that *cpa6* knockdown zebrafish are less sensitive to GABA-A antagonism (the theorized mechanism of action of PTZ) and non-selective muscarinic agonism via pilocarpine, suggests the resistance of the neuronal networks to stimulus input from different receptors, and by extension through different functional input signals.

We theorized that the most likely mechanism by which genetic ablation of *cpa6* alters neuronal excitability is by altering the processing of neuropeptides. Based on this hypothesis, there are several explanations of why humans with mutations in *CPA6* that reduce enzyme activity in the extracellular matrix are associated with elevated seizures while zebrafish embryos with knockdown of *cpa6* show reduced behavioral response to the proconvulsant stimulants PTZ and pilocarpine. One possible explanation is that CPA6 acts on different peptides in humans and fish because of the lack of conservation of the peptides. For example, natriuretic peptides do not have conserved C-terminal residues in humans and zebrafish [40, 41]. Similarly, peptides produced from proSAAS (*pcsk1nl*) are poorly conserved between zebrafish and mammals [42]. Thus, the pool of neuropeptides processed by CPA6 is likely to differ between fish and mammals. It is also possible that circuitry is different, leading to different patterns of innervation from the neurons releasing peptides processed by CPA6. Another consideration is that a point mutation is not necessarily the same as a knockdown. Our working assumption is that the point mutations associated with seizure disorders produced a loss-of-function phenotype, which should be mimicked by mRNA knockdown. This assumption is based on the extensive characterization of epilepsy/seizure-associated mutations in humans, which indicates that all disease-associated variants decrease the levels of CPA6 activity in the ECM *in vitro*, while a subset abolishes detectable expression of the mature form of the enzyme [9, 12, 13]. However, it is possible that the various point mutations all contribute to a gain-of-function phenotype, which would not be mimicked by *cpa6* knockdown.

Peptide precursor mRNAs are correlated with peptidergic neuronal firing, and the broad decrease in peptidergic precursor mRNAs and *cpe* mRNA in *cpa6* knockdown animals is suggestive of a reduction in the high frequency firing patterns that release neuropeptides (Fig 4). The reductions in the NMDA receptor subunit *grin1a*, the glutamate transporter *slc1a2b* and the activity-regulated transcript *cfos* suggest a depression of activity throughout the nervous system as well which may explain the resistance to stimulation by PTZ and pilocarpine. As a complement to this approach, we examined a time course of gene induction during stimulation with PTZ (Fig 5) and pilocarpine (Fig 6). While several mRNAs encoding neuropeptides increase acutely following PTZ treatment, these same mRNAs decrease 24 h after stimulation with pilocarpine indicating a second phase of the transcriptional program after pilocarpine treatment that does not occur after PTZ treatment. The finding that *cpa6* decreases during at least one time point after stimulation by both PTZ and pilocarpine also establishes that *cpa6* mRNA is regulated by neuronal activity. The finding that *cpe* mRNA generally remains unaltered despite being required in all dense core peptidergic granules suggests that this paradigm does not broadly release all granules. Chromogranin A (*Chga*), which is highly expressed in many peptidergic neurons throughout the vertebrate nervous system increases acutely with PTZ, but remains unchanged following pilocarpine treatment, further reinforcing that a subset of peptidergic neurons experience long term changes in their genetic program, likely due to high frequency firing within that population of cells. For pilocarpine, there is a mild increase in mRNAs encoding the precursors of excitatory neuropeptides neurotensin (*nts*) and endothelin 1 (*edn-1*), with long-term decreases in most peptides examined, suggesting a two-phase transcriptional program consistent with the pilocarpine model of seizure kindling in rodents [28, 29, 43]. Of note, injection of endothelin 1, a potent vasoconstrictor, causes ischemia induced seizures in rats [34], and the induction of this gene in the early phase of both stimulation paradigms is consistent with its role in excitatory transmission upstream of vascular and oxidative events, and observed increases in the active peptide after seizures [44, 45]. The C-terminal tryptophan residue of endothelin-1 is predicted to be a good CPA6 substrate [6, 7], is conserved from humans to zebrafish, and is required for receptor binding [46]. BDNF has been shown to be induced in many models of neuronal activation, and signaling through its receptor, TrkB, is thought to be important in potentiating this activity, especially in the temporal lobe [47].

The effects of PTZ are mostly transient for the genes examined in the current study, while pilocarpine induces long-lasting genetic changes, presumably reflecting differences in the stimulation of neuronal networks. In rodents, systemic administration of pilocarpine induces limbic seizures that become secondarily generalized. These events are followed by a latent “seizure free period” before the animal progresses to spontaneous seizures [28, 29, 48, 49]. Based on the observation that pilocarpine is capable of inducing long term changes in networks of excitability, the neurochemical changes in mRNA levels 24 h post-stimulation may partially explain long-term plastic changes in brain activity downstream of M1 activation. The finding that pilocarpine pre-treated embryos are resistant to PTZ treatment (Fig 7) supports the idea of a refractory period where the brain is less susceptible to excitability to GABA-A antagonism following a plasticity induced by muscarinic agonism. The transcriptional program present in this evoked state may be similar to the basal state of the *cpa6* knockdown animals, at least with regards to being resistant to excitatory stimulation. In summary, the present work strongly supports the presence of aberrant peptidergic signaling and suppression of excitatory inputs following *cpa6* loss of function or pilocarpine administration in zebrafish. Previous studies demonstrated the usefulness of rodent PTZ models for anti-epileptic drug discovery [50–52] and analysis of seizure generating mechanisms [53–55]. Future analysis may narrow down the pathways that are aberrantly activated or de-activated when extracellular CPA6 activity is reduced by gene mutations. The ultimate goal is to identify the neuropeptides that cause altered excitability and ultimately seizure susceptibility, which may be useful therapeutics.

## Supporting Information

S1 Fig Morpholino knockdown of *cpa6* mRNA in zebrafish embryos. Morpholino oligonucleotides were designed to block splicing of either exon 2 (CPA6-MO2) or exon 4 (CPA6-MO4). Splicing events are indicated by solid lines drawn between exons. Binding of the morpholino oligonucleotide results in exon exclusion (gray dashed lines) and frameshifts in the transcript. Only the first 6 exons of CPA6 are shown. The initiation methionine (“N-term”), propeptide cleavage site, and two of the critical zinc-binding residues (H69 and E71, based on numbering system of the active form of bovine carboxypeptidase A1) are shown.
(TIFF)

S2 Fig Effects of overexpression of *cpa6* mRNA on behavior in 3 dpf zebrafish embryos after PTZ and pilocarpine exposure. (A) Animals were tested for convulsive swimming behavior in the presence of 2.5 mM PTZ. Injection of *cpa6* mRNA had no significant effect on PTZ-evoked behaviors. Statistical analysis was performed by repeated measures ANOVA. (B) Animals were tested for number of crossings (locomotor activity) in the presence of 15 mM pilocarpine. No significant differences were observed between zebrafish injected with Ctrl-MO and zebrafish injected with 500 pg of *cpa6* mRNA. Statistical analysis was performed by Student’s *t* test. (C) Quantitative PCR revealed that injections of 500 pg or 1 ng of *cpa6* mRNA caused a ~20 and ~40-fold overexpression of cpa6 mRNA, respectively (n = 4). Statistical analysis was performed by one-way ANOVA, followed by Tukey HSD: ***, *p* < 0.001 compared with 0 pg group; #, *p* < 0.05 compared with 500 pg group. Error bars show SEM.
(TIFF)

S3 Fig Effects of CPA6-MO4 on convulsive swimming behavior in 3 dpf zebrafish embryos after 15 mM PTZ exposure. (A) Animals were tested for abnormal movement in the presence of 15 mM PTZ. After a baseline period animals were exposed to 15 mM PTZ. CPA6-MO-injected animals showed no difference in PTZ-evoked behaviors relative to the control-injected animals (Ctrl-MO). The injection of *cpa6* mRNA together with CPA6-MO4 was also not significantly different in this assay from Ctrl-MO or CPA6-MO-injected animals. Statistical analysis was performed by repeated measures ANOVA. (B) Latency to the first bout of convulsive swimming behavior was measured after 15 mM PTZ exposure. There was no significant difference between Ctrl-MO, CPA6-MO4 and CPA6-MO4 + mRNA injected embryos. Statistical analysis was performed by one-way ANOVA. Error bars show SEM.
(TIFF)

S4 Fig Comparison of quadrant transition and track length measurements for zebrafish embryos treated with 60 mM pilocarpine. Animals were treated with 60 mM pilocarpine and their behavior recorded on video with the same set-up used for Figs 1, 2, and 7. In this experiment, one group of zebrafish was injected with CPA6-MO2 and the other with Ctrl-MO; both groups were tested at 3 dpf. The videos were analyzed by an investigator blinded to the treatment group. (A) The videos were manually scored for the number of crossings between quadrants (locomotor activity). After a baseline period during which no movement was observed, 3 dpf zebrafish exposed to 60 mM pilocarpine showed increase number of crossings, this effect was reduced in zebrafish embryos injected with CPA6-MO2. (B) Computer analysis of the same videos using software that measured track length (mm). Statistical analysis was performed by Student’s *t* test: *, *p* < 0.05; **, *p* < 0.01 compared with respective Ctrl-MO group. Error bars show SEM (n = 18).
(TIFF)

S5 Fig Effects of *cpa6* knockdown on behavior in 3 and 7 dpf zebrafish embryos after PTZ exposure. (A) Animals were tested for convulsive swimming behavior in the presence of 2.5 mM PTZ. After a baseline period, CPA6-MO2 and CPA6-MO4 injected animals (3 dpf) exposed to 2.5 mM PTZ showed fewer bouts of convulsive swimming behavior, relative to Ctrl-MO. Statistical analysis was performed by repeated measures ANOVA followed by Tukey HSD: *, *p* < 0.05; **, *p* < 0.01 compared with Ctrl-MO group in respective time points; #, *p* < 0.05 compared with CPA6-MO2 group in respective time point. (B) Quantitative PCR revealed near total knockdown of the targeted exon for CPA6-MO2 (n = 4). Student’s *t* test: ***, *p* < 0.001 compared with Ctrl-MO group. (C) Quantitative PCR revealed near total knockdown in the targeted exon for CPA6-MO4 (n = 4). Student’s t test: ***, *p* < 0.001 compared with Ctrl-MO group. (D) CPA6-MO2 and CPA6-MO4 injected animals (7 dpf) exposed to 2.5 mM PTZ showed fewer bouts of convulsive swimming behavior, relative to Ctrl-MO. Statistical analysis was performed by repeated measures ANOVA followed by Tukey HSD: *, *p* < 0.05; **, *p* < 0.01; ***, *p* < 0.001 compared with Ctrl-MO group in respective time points; #, *p* < 0.05 compared with CPA6-MO2 group in respective time points. (E) Quantitative PCR revealed near total knockdown in the targeted exon for CPA6-MO2 (n = 4). Student’s t test: **, *p* < 0.01 compared with Ctrl-MO group. (F) Quantitative PCR revealed near total knockdown in the targeted exon for CPA6-MO4 (n = 4). Student’s t test: ***, *p* < 0.001 compared with Ctrl-MO group Error bars show SEM.
(TIFF)

S1 Table Primers used for qPCR assay. F, Forward; R, reverse.
(TIFF)
