# Supplementary figures and images for: Knockdown of Carboxypeptidase A6 in Zebrafish Larvae Reduces Response to Seizure-Inducing Drugs and Causes Changes in the Level of mRNAs Encoding Signaling Molecules
Source: PLoS One. 2016 Apr 6;11(4):e0152905. doi: 10.1371/journal.pone.0152905 (PMC4822968; doi:10.1371/journal.pone.0152905)

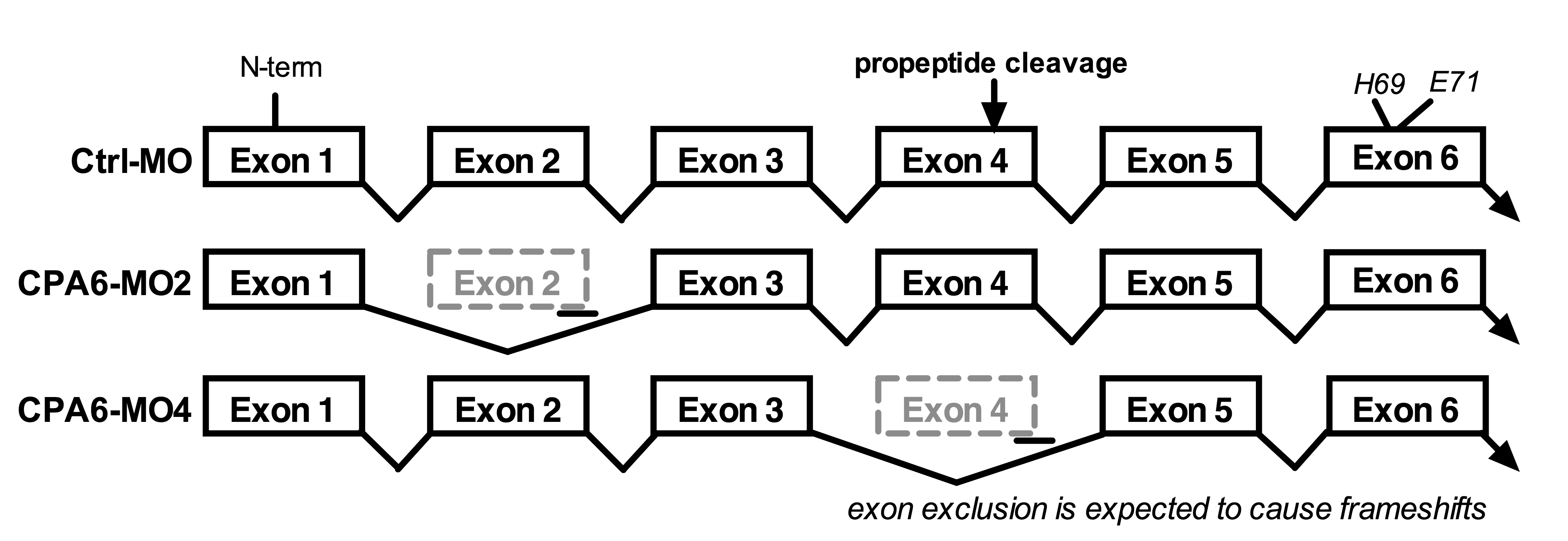

Supplement: S1 Fig — Morpholino oligonucleotides were designed to block splicing of either exon 2 (CPA6-MO2) or exon 4 (CPA6-MO4). Splicing events are indicated by solid lines drawn between exons. Binding of the morpholino oligonucleotide results in exon exclusion (gray dashed lines) and frameshifts in the transcript. Only the first 6 exons of CPA6 are shown. The initiation methionine (“N-term”), propeptide cleavage site, and two of the critical zinc-binding residues (H69 and E71, based on numbering system of the active form of bovine carboxypeptidase A1) are shown. (TIFF) [file pone.0152905.s001.tiff]

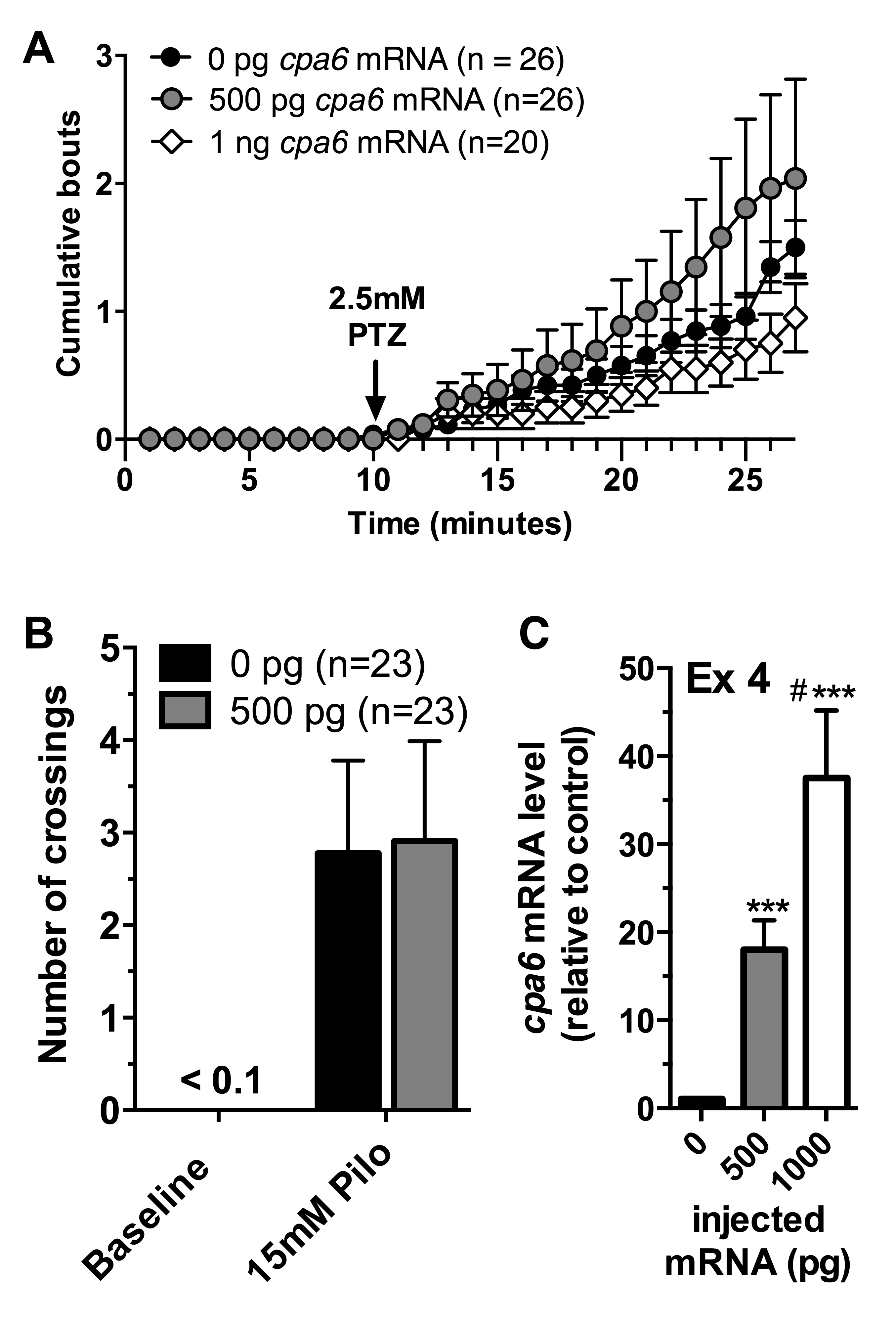

Supplement: S2 Fig — (A) Animals were tested for convulsive swimming behavior in the presence of 2.5 mM PTZ. Injection of cpa6 mRNA had no significant effect on PTZ-evoked behaviors. Statistical analysis was performed by repeated measures ANOVA. (B) Animals were tested for number of crossings (locomotor activity) in the presence of 15 mM pilocarpine. No significant differences were observed between zebrafish injected with Ctrl-MO and zebrafish injected with 500 pg of cpa6 mRNA. Statistical analysis was performed by Student’s t test. (C) Quantitative PCR revealed that injections of 500 pg or 1 ng of cpa6 mRNA caused a ~20 and ~40-fold overexpression of cpa6 mRNA, respectively (n = 4). Statistical analysis was performed by one-way ANOVA, followed by Tukey HSD: ***, p < 0.001 compared with 0 pg group; #, p < 0.05 compared with 500 pg group. Error bars show SEM. (TIFF) [file pone.0152905.s002.tiff]

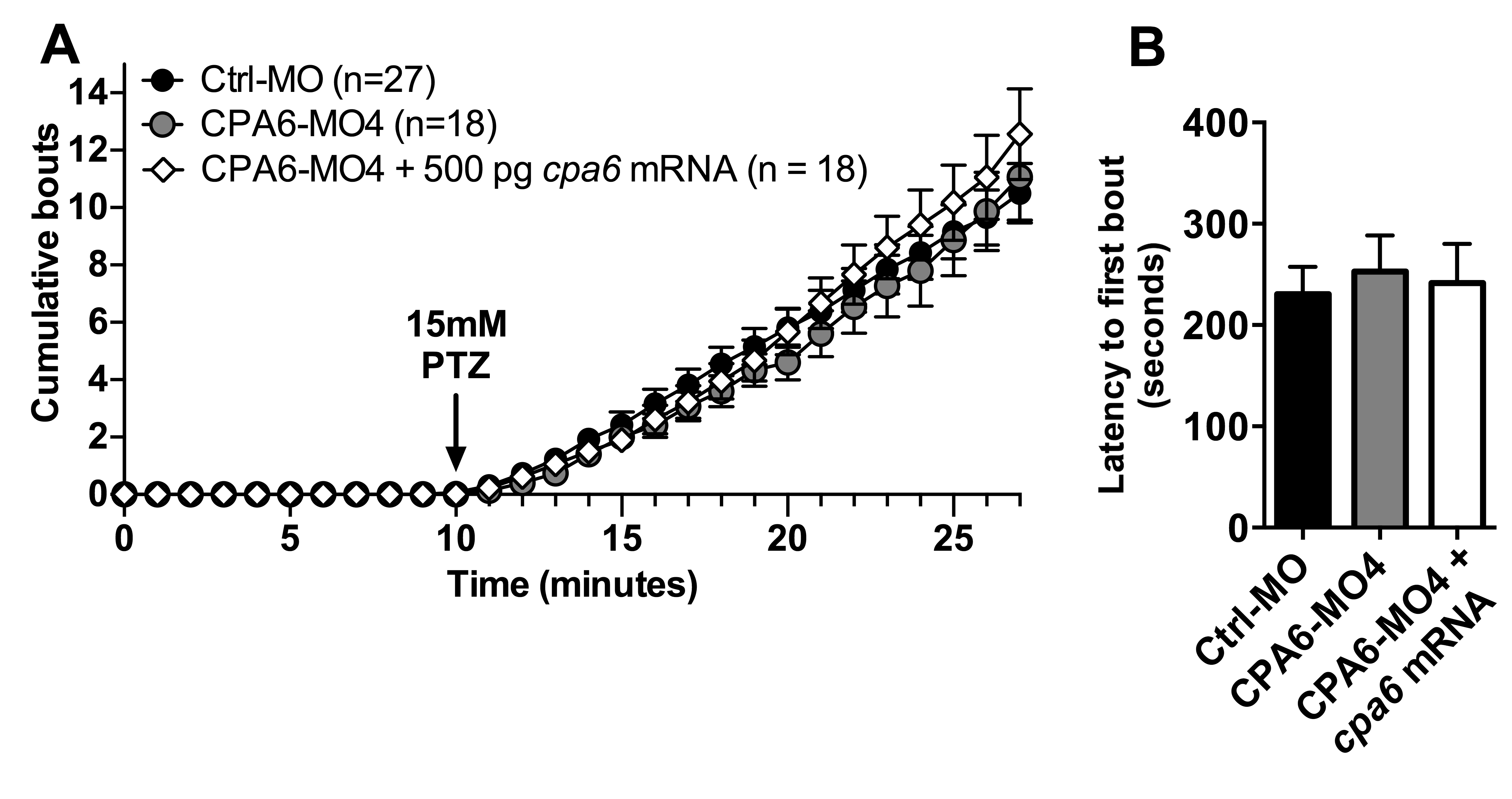

Supplement: S3 Fig — (A) Animals were tested for abnormal movement in the presence of 15 mM PTZ. After a baseline period animals were exposed to 15 mM PTZ. CPA6-MO-injected animals showed no difference in PTZ-evoked behaviors relative to the control-injected animals (Ctrl-MO). The injection of cpa6 mRNA together with CPA6-MO4 was also not significantly different in this assay from Ctrl-MO or CPA6-MO-injected animals. Statistical analysis was performed by repeated measures ANOVA. (B) Latency to the first bout of convulsive swimming behavior was measured after 15 mM PTZ exposure. There was no significant difference between Ctrl-MO, CPA6-MO4 and CPA6-MO4 + mRNA injected embryos. Statistical analysis was performed by one-way ANOVA. Error bars show SEM. (TIFF) [file pone.0152905.s003.tiff]

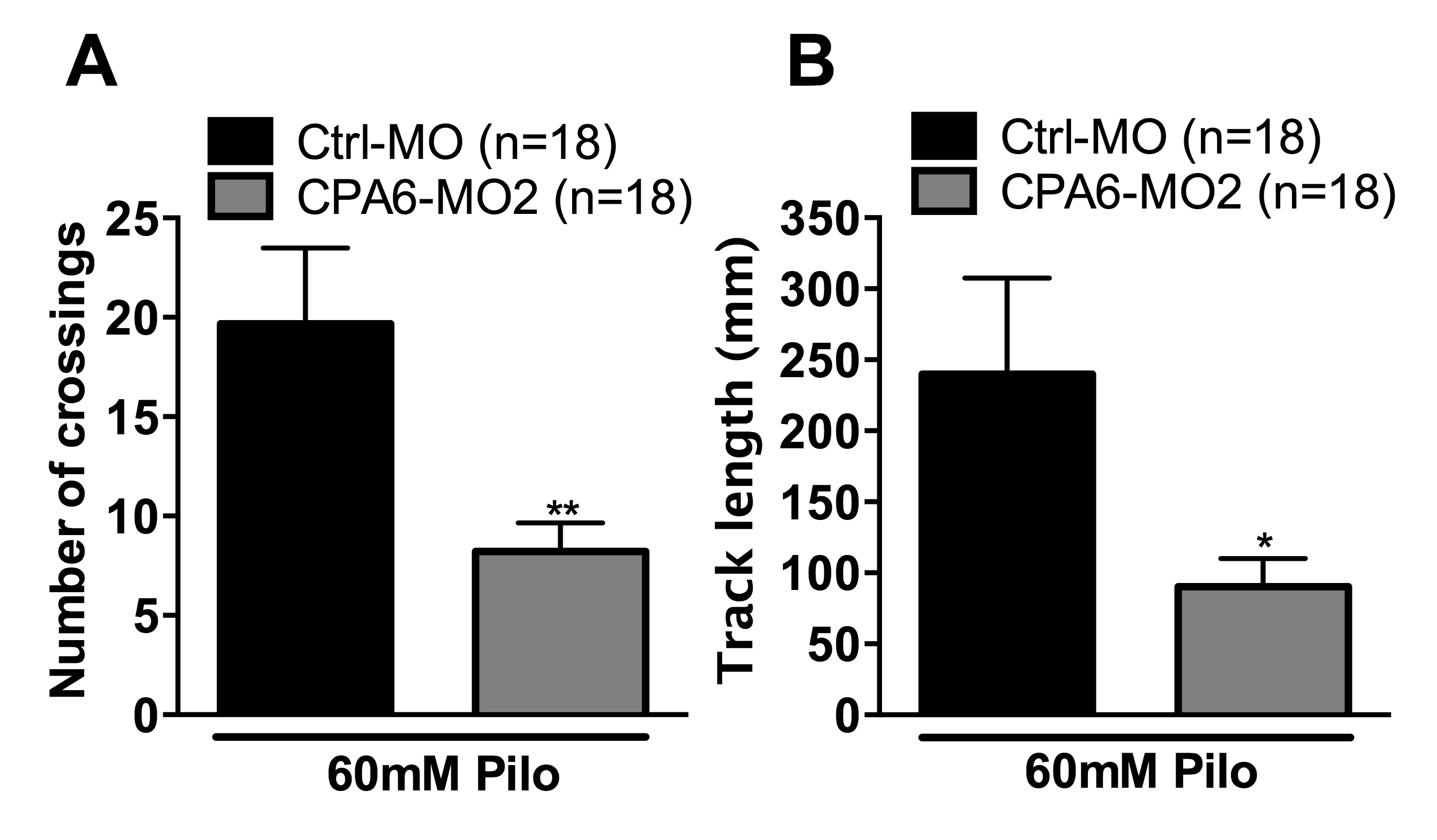

Supplement: S4 Fig — Animals were treated with 60 mM pilocarpine and their behavior recorded on video with the same set-up used for Figs 1, 2, and 7. In this experiment, one group of zebrafish was injected with CPA6-MO2 and the other with Ctrl-MO; both groups were tested at 3 dpf. The videos were analyzed by an investigator blinded to the treatment group. (A) The videos were manually scored for the number of crossings between quadrants (locomotor activity). After a baseline period during which no movement was observed, 3 dpf zebrafish exposed to 60 mM pilocarpine showed increase number of crossings, this effect was reduced in zebrafish embryos injected with CPA6-MO2. (B) Computer analysis of the same videos using software that measured track length (mm). Statistical analysis was performed by Student’s t test: *, p < 0.05; **, p < 0.01 compared with respective Ctrl-MO group. Error bars show SEM (n = 18). (TIFF) [file pone.0152905.s004.tiff]

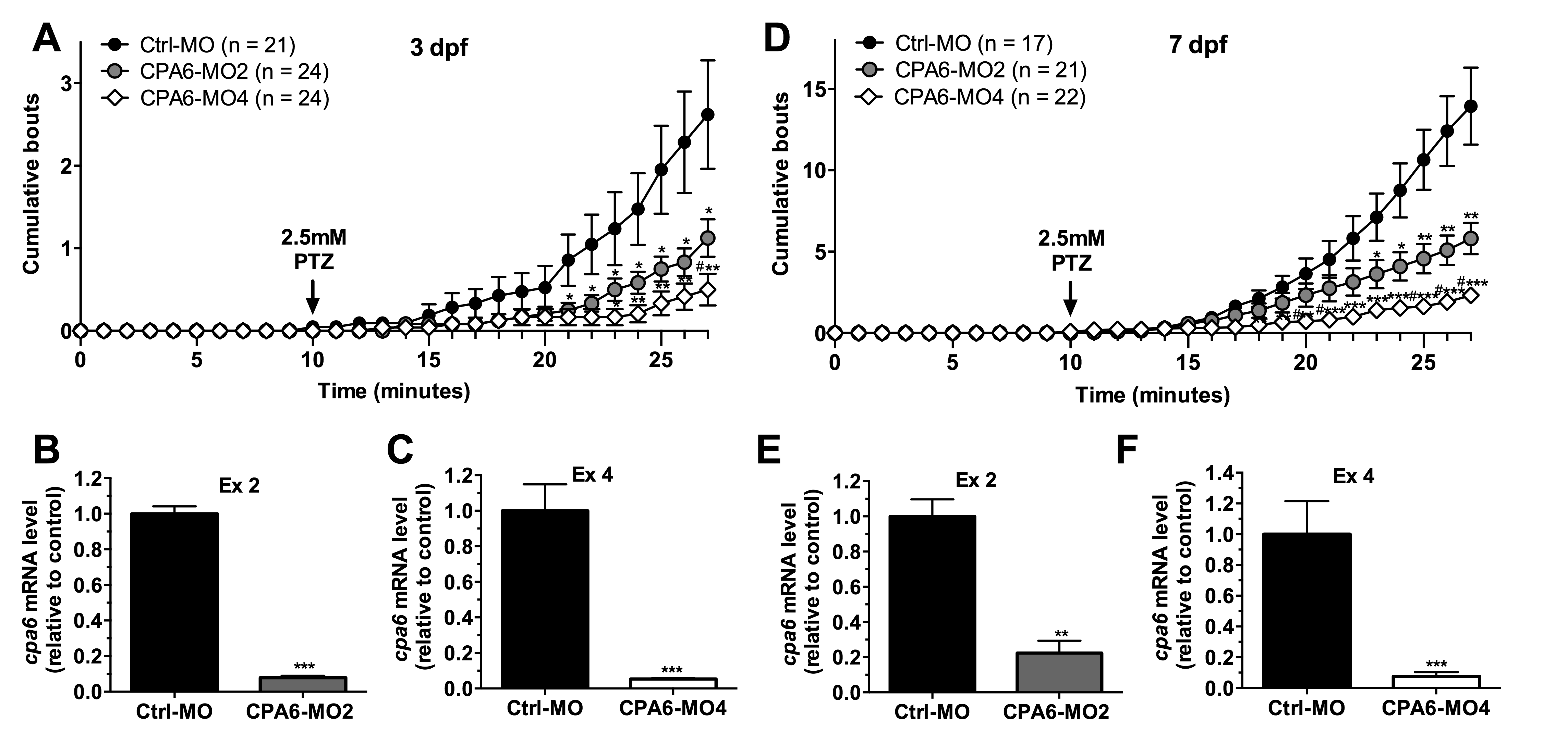

Supplement: S5 Fig — (A) Animals were tested for convulsive swimming behavior in the presence of 2.5 mM PTZ. After a baseline period, CPA6-MO2 and CPA6-MO4 injected animals (3 dpf) exposed to 2.5 mM PTZ showed fewer bouts of convulsive swimming behavior, relative to Ctrl-MO. Statistical analysis was performed by repeated measures ANOVA followed by Tukey HSD: *, p < 0.05; **, p < 0.01 compared with Ctrl-MO group in respective time points; #, p < 0.05 compared with CPA6-MO2 group in respective time point. (B) Quantitative PCR revealed near total knockdown of the targeted exon for CPA6-MO2 (n = 4). Student’s t test: ***, p < 0.001 compared with Ctrl-MO group. (C) Quantitative PCR revealed near total knockdown in the targeted exon for CPA6-MO4 (n = 4). Student’s t test: ***, p < 0.001 compared with Ctrl-MO group. (D) CPA6-MO2 and CPA6-MO4 injected animals (7 dpf) exposed to 2.5 mM PTZ showed fewer bouts of convulsive swimming behavior, relative to Ctrl-MO. Statistical analysis was performed by repeated measures ANOVA followed by Tukey HSD: *, p < 0.05; **, p < 0.01; ***, p < 0.001 compared with Ctrl-MO group in respective time points; #, p < 0.05 compared with CPA6-MO2 group in respective time points. (E) Quantitative PCR revealed near total knockdown in the targeted exon for CPA6-MO2 (n = 4). Student’s t test: **, p < 0.01 compared with Ctrl-MO group. (F) Quantitative PCR revealed near total knockdown in the targeted exon for CPA6-MO4 (n = 4). Student’s t test: ***, p < 0.001 compared with Ctrl-MO group Error bars show SEM. (TIFF) [file pone.0152905.s005.tiff]

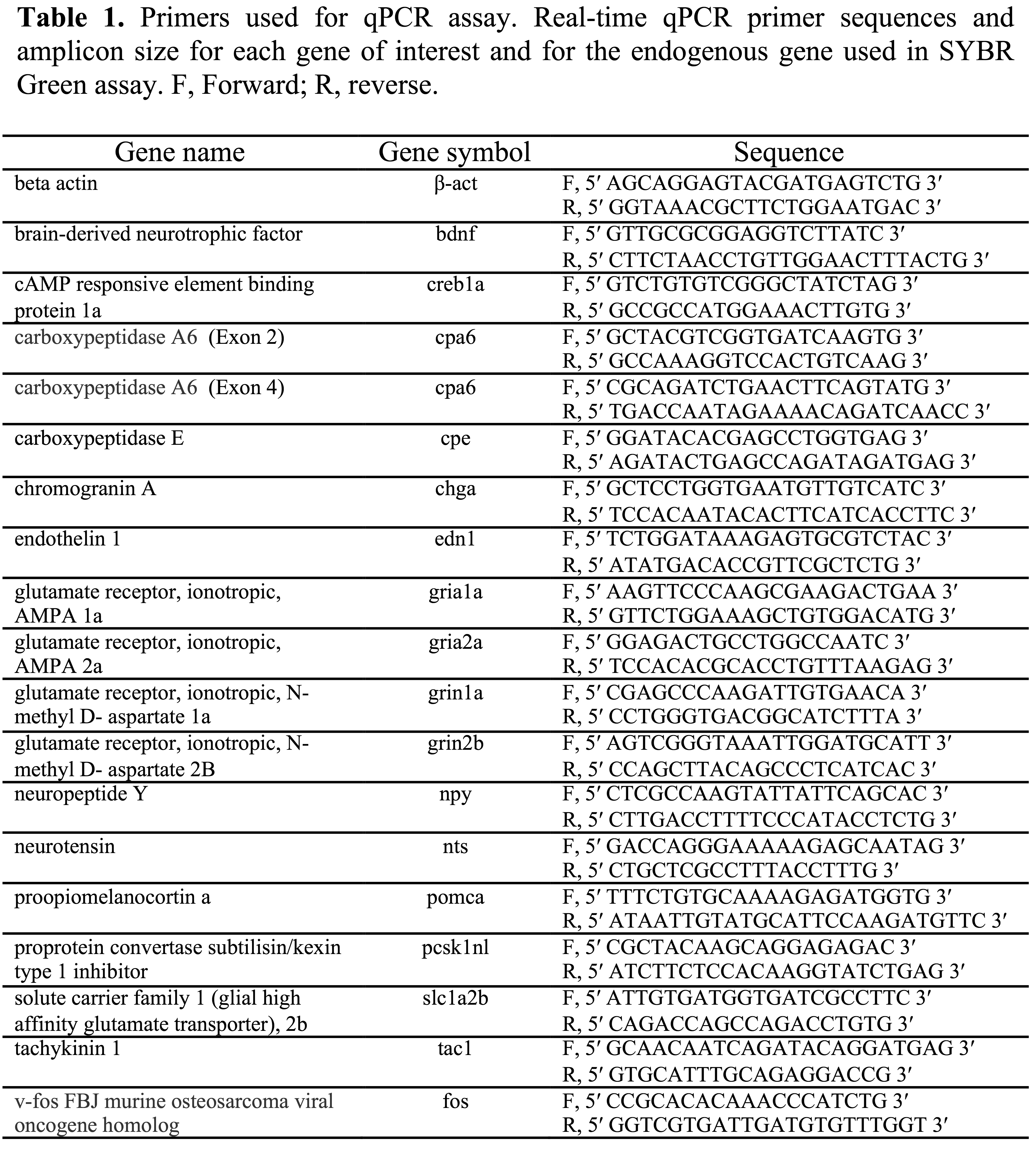

Supplement: S1 Table — F, Forward; R, reverse. (TIFF) [file pone.0152905.s006.tiff]
